# Citation Accuracy Challenges Posed by Large Language Models

**DOI:** 10.2196/72998

**Published:** 2025-04-02

**Authors:** Manlin Zhang, Tianyu Zhao

**Affiliations:** 1 Department of Obstetrics and Gynecology, Shengjing Hospital of China Medical University, Shenyang, China; 2 Department of Science and Technology Studies, University College London, Gower Street, London, WC1E 6BT, United Kingdom, 86 13674280942

**Keywords:** chatGPT, medical education, Saudi Arabia, perceptions, knowledge, medical students, faculty, chatbot, qualitative study, artificial intelligence, AI, AI-based tools, universities, thematic analysis, learning, satisfaction, LLM, large language model

 Large language models (LLMs) such as DeepSeek, ChatGPT, and ChatGLM have significant limitations in generating citations, raising concerns about the quality and reliability of academic research. These models tend to produce citations that are correctly formatted but fictional in content, misleading users and undermining academic rigor. In the recent study titled “Perceptions and earliest experiences of medical students and faculty with ChatGPT in medical education: qualitative study,” the section addressing concerns about ChatGPT deserves a deeper discussion [1].

There are several reasons for the citation issues in LLMs, which can be analyzed as follows. First, most LLMs cannot access paid subscription databases and therefore solely rely on open-access resources [2]. This limits the citations generated by LLMs to open-access journals, potentially omitting more significant research published in subscription-based journals. Second, LLMs are trained on vast amounts of text data and generate content by analyzing patterns and structures in text. However, they lack the ability to understand the content of the text or think critically, implying that they cannot judge the accuracy and reliability of information. Third, the algorithms underlying LLMs are often opaque, leaving users unable to understand the specific processes of information handling. This makes it difficult for users to determine the reliability of citations generated by LLMs and to effectively evaluate their results. Recent research also stated that half of generated search results lack citations, and only 75% of those with citations support the claims, posing trust concerns as user reliance grows[3].

Recently, an experiment conducted by the Journal of Clinical Anesthesia involved publishing a fictional article titled “Spinal Cord Ischemia After ESP Block” to test the spread and citation of a fabricated academic content. Surprisingly, the fictional article was widely cited, over 400 times, including in some journals with high impact factors[4], revealing a lack of rigor in academic citation practices, where many authors may not check the original literature and instead copy references directly. This incident sparked widespread discussion about academic citation practices, emphasizing the importance of critical thinking by scholars while citing materials.

The use of fictional citations by LLMs poses a multifaceted problem: it misleads users into drawing incorrect conclusions and making inappropriate decisions, undermines the rigor and credibility of academic research, and hinders the dissemination of knowledge by limiting access to accurate scientific information [5]. The issue of LLMs generating fictional citations is complex and requires the combined efforts of multiple stakeholders for resolution. Developers must continuously improve the LLM technology and algorithms, users must increase their awareness and critical evaluation skills while using LLMs, and academic institutions must strengthen the management and education in academic practices. Only through these efforts can we ensure that LLMs play a positive role in academic research and promote the dissemination and progress of knowledge.

## Abbreviations

LLM: large language model
